# Levonorgestrel release rates measured through analysis of two-rod contraceptive explants

**DOI:** 10.1016/j.conx.2020.100039

**Published:** 2020-08-21

**Authors:** Rachael Fuchs, Douglas Taylor, David W. Jenkins, Vivian Brache, Diane Luo, Laneta J. Dorflinger, Markus J. Steiner

**Affiliations:** a FHI 360, 359 Blackwell St. Suite 200, Durham, NC 27701, USA; b Asociación Dominicana Pro Bienestar de la Familia, Inc. (PROFAMILIA), Santo Domingo, Dominican Republic

**Keywords:** Implant, Sino-implant (II), Jadelle, Dominican Republic, China

## Abstract

**Objective:**

The objective was to characterize and compare in vivo rates of levonorgestrel (LNG) release from Sino-implant (II) and Jadelle® contraceptive implants.

**Study design:**

We sampled 48 Sino-implant (II) and 49 Jadelle® explant sets for residual LNG content from participants treated for up to 51 months in a randomized contraceptive efficacy trial in the Dominican Republic (DR). Additional Sino-implant (II) explants were obtained from 8 women who became pregnant in the DR trial and 10 who contributed 3 to 5 years of use in a cohort study in China. Baseline LNG loads were estimated from five unused implant sets per device type. Release profiles were estimated using mixture models that captured initial burst fractions and compared with efficacy and pharmacokinetics data from the DR trial.

**Results:**

Estimated baseline LNG loads for Sino-implant (II) and Jadelle® were 142.8 mg and 150.5 mg, respectively (vs. the labeled 150 mg). There was an initial burst release of drug (5.6% and 7.9%, respectively) followed by an exponential decrease in LNG content evident for each device. Release rates were significantly lower for Sino-implant (II) throughout the treatment period, with estimated rates after 3 years of 24.2 mcg/day and 29.0 mcg/day for Sino-implant (II) and Jadelle®, respectively. The estimated Sino-implant (II) rate after 3 years was similar to the predicted rate after 5 years (23.6 mcg/day) for Jadelle® (rate ratio: 1.03; 95% confidence interval: 0.92–1.13).

**Conclusions:**

Sino-implant (II) LNG release rates were significantly lower than Jadelle® with Sino-implant (II) rates through year 3 comparable to Jadelle® rates through year 5. These results reinforce the 3-year duration of action for which Sino-implant (II) was prequalified by the World Health Organization.

**Implications:**

This analysis confirms the WHO prequalification of Sino-implant (II) for 3 years of use and supports different durations of action for Jadelle® and Sino-implant (II). It provides additional evidence that this approach can complement efficacy trials in determining duration of action of hormonal contraceptives in general.

## 1 Introduction

Sino-implant (II) is a two-rod subdermal contraceptive implant system developed in China in the 1980s and 1990s that is approved for 4 years of continuous use by the China Food and Drug Administration. The device has a similar design and nominal 150-mg loading dose (75 mg/rod) of levonorgestrel (LNG) as Jadelle® (Bayer Oy, Finland), which is approved for 5 years of use by the U.S. Food and Drug Administration and other stringent regulatory authorities.

LNG release rates from Jadelle® are approximately 100 mcg/day 1 month after insertion and decrease to 40, 30 and 25 mcg/day, respectively, at the end of year 1, 3 and 5 of use [1,2]. Mean total (bound and unbound) serum LNG concentrations decline over the first 2 years of use, however, are comparatively stable between 3 and 5 years of use [3]. This apparent inconsistency between decreasing release rates and relatively stable total LNG concentrations between years 3 to 5 may be explained in part by changes in the fraction of total LNG that is bound with high affinity to sex hormone binding globulin (SHBG), bound with low affinity to albumin, and unbound or “free” (1%–2% of the total) as SHBG levels are impacted by LNG exposure [2,4,5]. Studies have also shown some inconsistency in pregnancy rates for Jadelle®. In studies that informed the label (*N* = 1393), the Pearl Index (pregnancies per 100 women-years) increased after year 4 (from below 0.2 to 0.9 in year 5) [1,6], while a more recent multicenter trial (*N* = 997) reported a Pearl Index of 0.4 in the first 3 years but no pregnancies in the last 2 years [7,8].

To support World Health Organization (WHO) prequalification of Sino-implant (II) [9], FHI 360 and its partners conducted an efficacy trial of *N* = 514 Sino-implant (II) users in the Dominican Republic (DR) [10]. The method proved highly effective over the first 3 years of use, with a cumulative Pearl Index of 0.18 [95% confidence interval (CI): 0.02–0.65]. The study was stopped early due to an unexpectedly high pregnancy rate in year 4 (3.54; 95% CI: 1.53–6.97), however, and Sino-implant (II) was ultimately prequalified as a 3-year method [11]. Sino-implant (II) is now distributed globally under the brand name Levoplant^TM^ (Shanghai Dahua Pharmaceutical Co., Ltd., China).

A pharmacokinetics (PK) substudy in the DR trial found geometric mean (GM) total plasma LNG concentrations that were similar between Sino-implant (II) and Jadelle® at year 1 (GM ratio: 0.94; 90% CI: 0.87–1.02) but 19%, 22% and 32% lower in the Sino-implant (II) group at years 2, 3 and 4 (p <.01 at each time point and for the trend across time) [10]. These results, combined with the absence of pregnancies in the relatively small (*N* = 136) cohort of Jadelle® users, provide compelling evidence that the period over which Sino-implant (II) remains highly effective is shorter than Jadelle® [10].

Prior to conducting the DR trial, four of the authors (M.S., D.T., D.J., L.D.) estimated and compared LNG release rates using sparse Sino-implant (II) data and historical reports for Jadelle® and concluded that the devices may have similar clinical performance through 3 years [12]. That exploratory analysis was limited because duration of implant use was not precisely known and there were no subject-level Jadelle® data available to the authors. Here we report on a more rigorous, comparative analysis of explant data to better inform the relative durations of effectiveness of the methods and the amount of LNG release associated with a high degree of contraceptive efficacy.

## 2 Material and methods

This is a secondary analysis of explant data obtained from two recent efficacy studies. Each was conducted following Good Clinical Practice guidelines and approved by the Protection of Human Subjects Committee at FHI 360 and applicable local institutional review boards. The majority of the data come from the 4:1 randomized study of Sino-implant (II) and Jadelle® in the DR [10]. Briefly, participants in that study had clinic visits scheduled 1, 6, 12, 18, 24, 30, 36, 42, 48, 51, 54, 57 and 60 months after device insertion, at which times pregnancy status was assessed and blood was drawn for total LNG and SHBG testing (the latter in a subset of users). Follow-up was truncated at 48 months for most women due to a higher-than-expected pregnancy rate in the fourth year of Sino-implant (II) use.

We selected explant sets from 105 DR participants [56 Sino-implant (II) and 49 Jadelle®] for testing. Sampling was based on a goal of obtaining one device (two explants removed from one woman) per group at each of study months 1 through 51. The target sample size of 51 explant sets per group was estimated to provide approximately 80% power to detect differences in LNG release profiles between the device groups at the .05 significance level. Power was estimated by simulating 2000 sets of data generated under a nonlinear model previously used to describe Jadelle® release rates [13] and available sparse Sino Implant (II) data [12]. A single device was randomly chosen for months with more than one available; for months with no data, the device removed closest in time was chosen. There were only six women with explants available in the Jadelle® group between months 39 and 47, and all were selected. We also selected the device removed closest to day 0 in each group and all Sino-implant (II) explants from eight women who became pregnant on or before the explant removal date.

A noncomparative cohort study in China evaluating the effectiveness of Sino-implant (II) [14] was a second source of data used to assess the effect of race on release profiles. Due to the small number of removals done at study clinics, explants were available for only 10 women in the China study. Removed explants from both studies were washed according to a cleaning protocol developed by the University of North Carolina Infectious Diseases Research Laboratory [12]. Explants were then shipped to Shanghai Dahua Pharmaceutical Co. (China) for LNG content testing using a validated high-performance liquid chromatography method for lot release testing that was approved by WHO as part of the Sino-implant (II) prequalification process^1^. An additional five unused implant sets of each type were tested for LNG loading dose using the same method.

A series of prespecified, empirical models was subsequently fit to the LNG content data for each device type, initially restricted to the DR data. Details of all four models can be found in the supplement to this paper. Briefly, we considered a monoexponential decay model, a monoexponential decay model with a burst fraction, a biexponential model and a nonlinear model previously used by the Population Council to describe release rates for Jadelle® [13]. Each model was fit to the LNG content data using the SAS/STAT® NLMIXED procedure [15], assuming independent and identically distributed multiplicative log-normal errors. Model results were compared using Bayesian information criteria to select the best model for each device type. Daily release rates were obtained by taking derivatives of the LNG content model with respect to time.

The model with the best fit for both devices was the monoexponential decay model with burst fraction:

(2) $$L_{i}=I\left(t_{i}=0\right)\cdot A+I\left(t_{i}>0\right)\cdot \left(1-f\right)\cdot A\cdot \exp\left(-kt_{i}\right),$$

where *L*_*i*_ is the measured LNG content for the *i*-th device, *k* is the exponential rate parameter, *A* is the baseline load of LNG, *t*_*i*_ is the time (in days) from insertion to removal (*t*_*i*_ = 0 for unused implants), *I*(·) is the indicator function and *f* is the fraction of LNG released in the initial burst.

Based on our analysis plan, data from the 10 women in the China study were only included in an updated Sino-implant (II) model if the results all fell within 2 standard deviations (SD) of predicted mean content levels. Data from the women who became pregnant while using Sino-implant (II) were used to graphically assess the extent to which release profiles may have differed for pregnant women, but were excluded when fitting the final model to avoid potential selection bias.

The difference in release profile between devices was assessed by including separate regression parameters for each device type and testing their significance at the .05 level. The impacts of body mass index (BMI), age and race were assessed by adding covariates to the regression parameters (excluding baseline load, which was assumed to be independent of covariate effects). Estimated mean LNG content and release rates from the final model were plotted over time, together with pointwise upper and lower fifth percentiles for LNG content and 95% CIs for release rates. Estimated release rates were contrasted with GM total plasma LNG concentrations (nmol/L), serum SHBG concentrations (nmol/L) and the free LNG index [FLI; the ratio of total LNG (nmol/L) to SHBG (nmol/L), times 100] using graphical methods.

We also compared LNG release profiles by calculating the similarity factor, a nonparametric statistic intended to compare in vitro drug dissolution profiles [16]. Details are provided in the supplemental materials.

## 3 Results

There were no meaningful differences in age, race or BMI between device groups among the women in the DR who contributed explant data (Table 1). Demographic characteristics of DR women selected for testing were also similar to the remainder of the DR cohort (results not shown). The 10 women from the China cohort had similar BMI as in the DR, but they were generally older (50% between 35 and 44 years of age compared to 0% in the DR) and were all Asian, whereas about 90% of women from the DR cohort were biracial.

**Table 1:** Demographics of women contributing to explant analysis from China and DR studies

	Sino-implant (II)		Jadelle®
	China (*N* = 10)	DR (*N* = 56)	DR (*N* = 49)
**Age (in years): *N* (%)**			
18–24	0 (0.0)	37 (66.1)	29 (59.2)
25–29	0 (0.0)	17 (30.4)	15 (30.6)
30–35	5 (50.0)	2 (3.6)	5 (10.2)
> 35	5 (50.0)	0 (0.0)	0 (0.0)
Mean (SD)	37.2 (3.7)	22.9 (3.4)	23.8 (4.0)
Min to max	33 to 42	18 to 31	18 to 32
**Race: *N* (%)**			
White	0 (0.0)	3 (5.4)	2 (4.1)
Biracial	0 (0.0)	48 (85.7)	47 (95.9)
Black	0 (0.0)	5 (8.9)	0 (0.0)
Asian	10 (100)	0 (0.0)	0 (0.0)
**BMI (kg/m**^**2**^**): *N* (%)**			
< 25	6 (60.0)	29 (51.8)	34 (69.4)
25–30	3 (30.0)	20 (35.7)	8 (16.3)
≥ 30	1 (10.0)	7 (12.5)	7 (14.3)
Mean (SD)	25.3 (3.2)	25.0 (4.5)	24.1 (4.6)
Min to max	20.4 to 31.2	16.1 to 35.6	16.7 to 34.5

After explant testing was complete, 13 explants assessed on the same day by a new lab technician were determined to be gross outliers (including coefficients of variation between rods that exceeded 60%). Consequently, all 15 explants tested by that technician on that day were excluded from analysis. To maintain study size, the excluded explants were replaced with ones randomly selected from the same device group, matched (± 3 months) on time to removal.

The monoexponential decay model with burst fraction provided the best fit to both the Sino-implant (II) and Jadelle® data. The China data were included in subsequent modeling since all content values fell within 2 SD of predictions obtained when excluding those data. LNG content values were outside the prediction bands for two of eight women who became pregnant while using Sino implant (II). Age, BMI and race were not significantly associated with LNG release profiles (see supplementary materials). Consequently, the final model only included effects for baseline LNG content, burst fraction and exponential release rate for each device type, with a common residual variance.

**Fig. 1:**
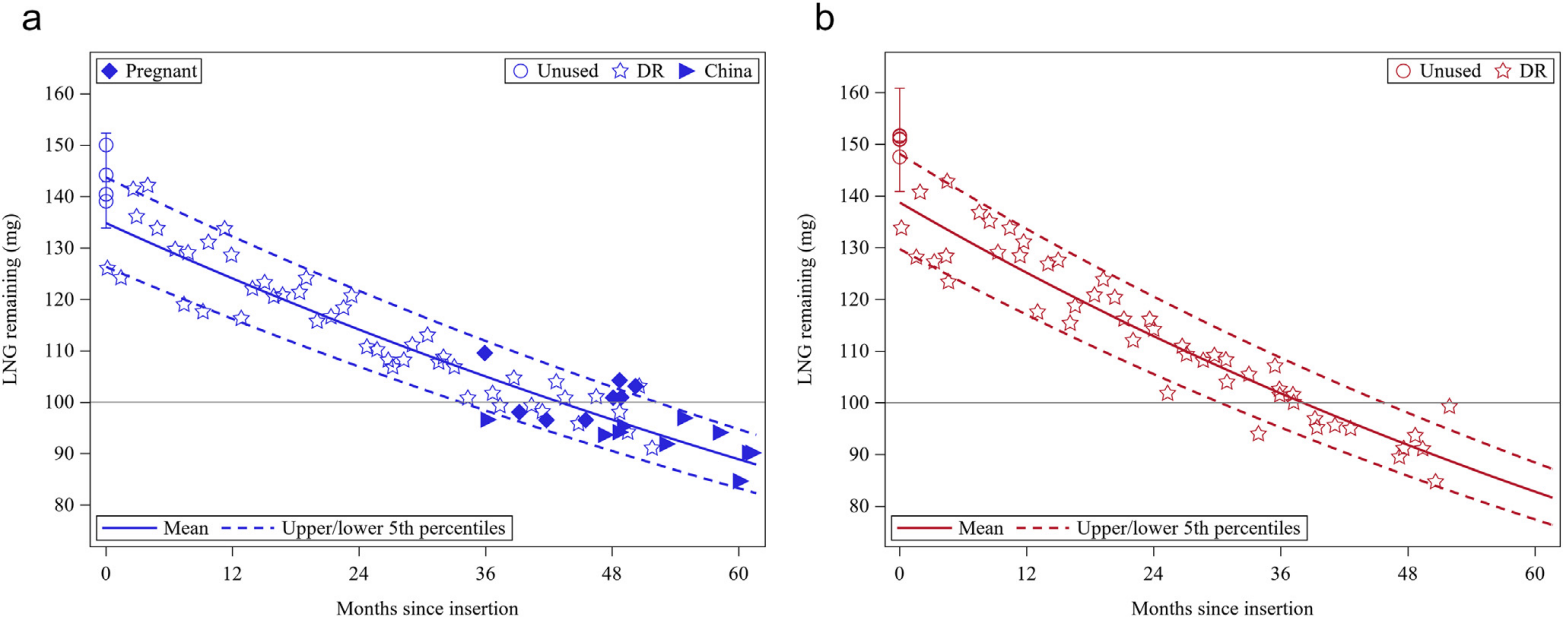
Observed and estimated LNG content remaining in Sino-implant (II) [a] and Jadelle [b] explants, with estimated upper and lower fifth percentiles (data from eight pregnant women was not used in model fit).

**Fig. 2:**
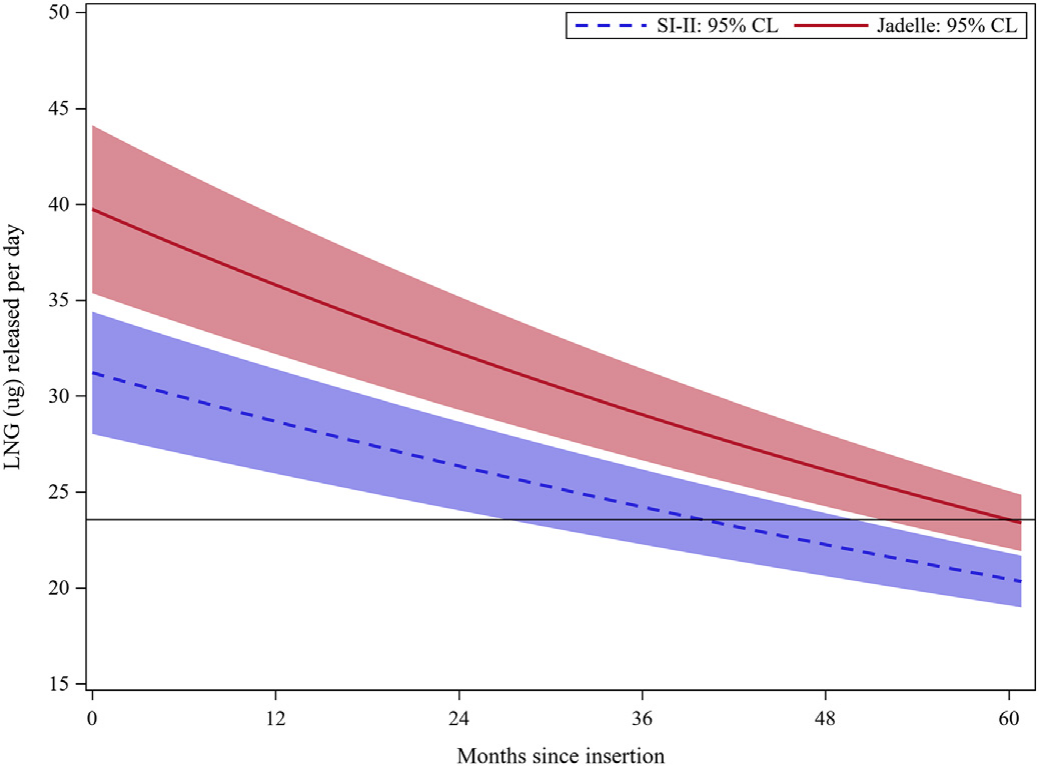
Estimated release rates with 95% CIs (horizontal line is predicted year 5 rate for Jadelle®)

Observed and estimated mean residual LNG content values are plotted over time in Fig. 1A and B, and estimated daily release rates are presented in Fig. 2. Mean baseline LNG content was lower than 150 mg for Sino-implant (II) (142.8 mg vs. 150.5 mg for Jadelle®; p =.039) but remained within product specifications^2^. Estimated burst fractions were similar between devices [5.7% of LNG content released in initial burst for Sino-implant (II) vs. 7.9% for Jadelle®; p =.42], but the exponential rate parameter was significantly smaller for Sino-implant (II) (0.00023 vs. 0.00029; p <.001). Daily release rates were likewise lower for Sino-implant (II) than Jadelle® throughout the assessment period (Table 2): 28.7 vs. 35.8 mcg/day (year 1), 26.4 vs. 32.2 (year 2), 24.2 vs. 29.0 (year 3) and 22.2 vs. 26.2 (year 4). This is consistent with approximately a 2-year shift over which the devices released similar amounts of LNG. Of note, the pregnancy Pearl Index for Sino-implant (II) reported in the parent DR trial increased significantly after year 3 (from below 0.5 to 3.5; Table 2), when the mean release rate had fallen below 24.2 mcg/day.

**Table 2:** Estimated LNG release rates^a^ (SE), rate ratios and pregnancy rates

	Release rate (mcg/day)			Pregnancy rates (95% CIs)	
Year of use	Sino-implant (II) (*N* = 58)	Jadelle® (*N* = 49)	Rate ratio (95% CI)	Sino-implant (II)^b^ (*N* = 514)	Jadelle® (*N* = 1393)^c^
1	28.7 (1.4)	35.8 (1.8)	0.80 (0.69–0.91)	0.00 (0.00–0.79)	0.08 (0.01–0.24)
2	26.4 (1.2)	32.2 (1.4)	0.82 (0.72–0.92)	0.28 (0.01–1.55)	0.10 (0.01–0.29)
3	24.2 (1.0)	29.0 (1.2)	0.83 (0.74–0.93)	0.34 (0.01–1.91)	0.13 (0.01–0.37)
4	22.3 (0.8)	26.2 (0.9)	0.85 (0.76–0.94)	3.54 (1.53–6.97)	0.00 (0.01–0.03)
5^d^	20.4 (0.7)	23.6 (0.7)	0.86 (0.79–0.95)	NA	0.88 (0.30–1.73)
Year 1 Sino-implant (II) vs. year 3 Jadelle®:			0.99 (0.86–1.11)		
Year 2 Sino-implant (II) vs. year 4 Jadelle®:			1.01 (0.89–1.12)		
Year 3 Sino-implant (II) vs. year 5 Jadelle®:			1.03 (0.92–1.13)		

a Rates are calculated at the end of the year of use.

b From study in the DR [6]. Includes two chemical pregnancies detected at the end of year 4.

c From Jadelle® prescribing information [5]; restricted to women < 36 years of age. No pregnancies were observed among 137 women randomized to Jadelle® in the study in the DR.

d Release rates for Jadelle® at year 5 are extrapolations beyond the range of the data.

**Fig. 3:**
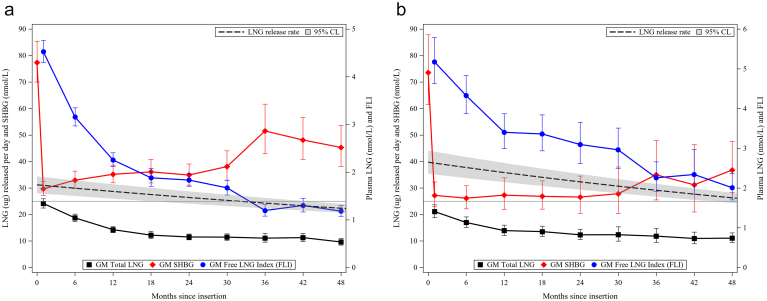
(A) Estimated Sino-implant (II) LNG release rate overlaid with geometric mean total LNG, SHBG and free LNG index from the efficacy study in the DR [6] (reference line at 25-mcg release rate). (B) Estimated Jadelle® LNG release rate overlaid with geometric mean total LNG, SHBG and free LNG index from the efficacy study conducted in the DR [6] (reference line at 25-mcg release rate).

We described the relationship between release rates and total LNG, SHBG and FLI by overlaying estimated release rates with GM total LNG, GM SHBG and GM FLI from the DR study (Fig. 3A and B). There was a rapid ~ 60% reduction from baseline SHBG concentrations in the first month after insertion of Sino-implant (II) followed by a gradual increase in SHBG through year 2.5 (from ~ 30 to 40 nmol/L) and a more substantial increase (to ~ 45–50 nmol/L) thereafter. The SHBG profile for Jadelle® was qualitatively similar but with lower and more stable levels between month 1 and year 2.5 (~ 25–30 nmol/L) and a more modest increase (to ~ 35–40 nmol/L) thereafter. In both device groups, SHBG concentrations began to rebound when the estimated LNG release rate fell below ~ 30 mcg/day. Total LNG concentrations decreased between years 1 and 4 but at a significantly faster rate for Sino-implant (II) (results not shown). The FLI uniformly decreased across the assessment period for both device types but much more rapidly in the first year of use.

## 4 Discussion

Efficacy studies of new long-acting contraceptive methods typically require a substantial investment of resources, including years of participant follow-up to precisely establish the duration of action of a method. For extended-release methods based on drugs with a well-characterized PK and pharmacodynamic relationship, studies which accurately characterize rate of drug release over time can complement — and potentially reduce the required scope of — large efficacy trials. We undertook an analysis of Sino-implant (II) and Jadelle® explant data to better understand LNG release profiles of the two devices and the relative durations over which the methods remain highly effective.

Estimated release rates were significantly lower for Sino-implant (II) than Jadelle®: ranging from 20% lower at year 1 to 15% lower at year 4, with approximately a 2-year shift in time when rates were the same. In particular, the estimated Sino-implant (II) release rate at year 3 of 24.2 mcg/day was comparable to the predicted 23.6 mcg/day for Jadelle® at the end of the latter’s 5-year intended duration of use (rate ratio: 1.03; 95% CI: 0.92–1.13). The 3-year Pearl Index observed for Sino-implant (II) in the parent trial (0.18; 95% CI: 0.02–0.65) was also comparable to the reported 5-year Pearl Index of 0.17 for Jadelle® [1]. These results support the conclusion that Sino-implant (II) has a shorter duration of action than Jadelle®. Although both implants are comprised of a similar silicone elastomeric matrix, differences in release rate could be associated with material properties such as LNG and excipient manufacturing sources, LNG particle size, LNG to matrix ratio, crosslink density of the matrix, inner core diameter and outer membrane thickness.

In contrast to a consistent 15%–20% lower LNG release rate estimated for Sino-implant (II), PK analysis in the parent DR trial observed steadily decreasing relative total LNG concentrations, ranging from 6% less than Jadelle® at year 1 to 32% less at year 4 [10]. This difference in trends may be due in part to an increase in SHBG concentrations, and changes in SHBG and albumin fractions (bound as well as free), as LNG exposure decreases. It is also possible that the monoexponential decay model with burst fraction failed to adequately capture nuances in LNG release profiles. The estimated release rates for Jadelle® reported here are similar to those in the historical literature for that device. However, they are markedly lower than were estimated in a recent population PK model of oral, intravenous, intrauterine and subdermal LNG administration [4]. Those authors estimated near zero-order release rates of 33.9, 33.2 and 32.8 mcg/day after 3, 4 and 5 years of Jadelle® use compared to 29.0, 26.2 and 23.6 mcg/day in our analysis. Alternative drug release models could be considered depending on the geometry of the implant system [17]. The challenge is that many of the parameters needed for their application, such as solubility, diffusion coefficients and partition coefficients, are not known with sufficient confidence to apply in the current setting.

A previous comparison of sparse Sino-implant (II) and historical Jadelle® data suggested that the LNG release rates might be comparable over the first 3 years of use based in part on an *f*2 similarity factor of 80.6 (90% CI: 70.8–85.7) [12] (values greater than 50 indicate similar release profiles in the context of in vitro dissolution studies for which the statistic was developed [16]). We observed a comparably high *f*2 of 78.7 (90% CI: 70.4–80.7) in the current study. However, the significant difference in modeled release rates between devices suggests that *f*2 was not an appropriate measure of agreement in our setting where explant data had to be grouped into 6-month use periods and the number of data points per time period did not meet the suggested guidance [18].

Strengths of our analysis include the use of explants collected in carefully implemented clinical trials with precise information on time of removal, covariate data and supportive information on plasma drug levels. The primary limitations were the low power to detect effects of race and the lack of a control group in the China study, for which we had limited explant data and only among women who had used Sino-implant (II) for more than 3 years.

The estimated relative LNG release rates described here are consistent with the clinical pregnancy data observed in the parent study of Sino-implant (II) and historical data for Jadelle®. In particular, a release rate of approximately 25 mcg/day is associated with a high degree of contraceptive efficacy. Characterizing the relationship between release rates and PK data (including total LNG, SHBG and free LNG) is made challenging due to dynamics of LNG exposure and inhibitory effect on SHBG and — besides simple descriptive comparisons — is beyond the scope of this paper. Nonetheless, our results lend confidence to the conclusion that Sino-implant (II) is similarly effective through 3 years of use as Jadelle® through year 5 and support the 3-year duration for which Sino-implant (II) was prequalified by WHO [11].

## Appendix A Supplementary data

Supplementary material Image 1

## Footnotes

1 The procedure used to quantify levonorgestrel remaining in the implants was a proprietary manufacturer method used for lot release testing and was approved by WHO as part of the Sino-implant (II) prequalification process (Ref. No. RH028, available from: https://extranet.who.int/prequal/medicine/3997). An alternate method for levonorgestrel quantification is the assay procedure found in the Chinese Pharmacopeia; China Food and Drug Administration. WS1-(X-281)-2004Z standard for Levonorgestrel Silastic Implants (II); 2004.

2 The original specification for levonorgestrel content is 90.0%–110.0% label claim based on China Food and Drug Administration, WS1-(X-281)-2004Z standard for Levonorgestrel Silastic Implants (II); 2004. The specification has been updated to 95.0%–105.0% label claim through the WHO prequalified status for the product.
