# Supplementary material for: Levonorgestrel release rates measured through analysis of two-rod contraceptive explants
Source: Contracept X. 2020 Aug 21;2:100039. doi: 10.1016/j.conx.2020.100039 (PMC7509190; doi:10.1016/j.conx.2020.100039)
Supplement: Supplementary file 1 — Supplementary material [file mmc1.docx]

Supplemental Materials for the manuscript titled

Levonorgestrel release rates measured through analysis of two-rod contraceptive explants

Rachael Fuchs^a,^^[[1]](#footnote-2)^, Douglas Taylor^a^, David W. Jenkins^a^, Vivian Brache^b^, Diane Luo^a^, Laneta J. Dorflinger^a^, Markus J. Steiner^a^,

Author affiliations:

^a^ FHI 360, 359 Blackwell Street Suite 200, Durham, NC 27701, USA

^b^ Asociación Dominicana Pro Bienestar de la Familia, Inc. (PROFAMILIA), Santo Domingo, Dominican Republic

Four pre-specified, empirical models were fit to the LNG content data for each device type. The simplest was a mono-exponential decay model previously used to assess LNG release from intra-uterine devices [1]:

$L_{i}=A\cdot\exp(-kt_{i}),$ (1)

where *L_i_* is the measured LNG content for the *i-th* device, *k* is the exponential rate parameter, *A* is the initial (baseline) load of LNG, and *t_i_* is the time (in days) from insertion to removal (*t_i_*=0 for unused implants). Because the PK of sub-dermal implants may be associated with an initial rapid release of drug [2], we also considered two mixture models to capture biphasic release effects. The first assumed a fraction of drug was immediately released upon device insertion:

$L_{i}=I(t_{i}=0)\cdot A+I(t_{i}>0)\cdot(1-f)\cdot A\cdot\exp(-kt_{i}),$ (2)

where *I*(·) is the indicator function and *f* is the burst fraction. The second assumed a bi-exponential process with both a fast and a slow release component:

$L_{i}=f\cdot A\cdot\exp(-k_{f}t_{i})+(1-f)\cdot A\cdot\exp(-kt_{i}),$ (3)

where *k_f_* and *k* are the exponential rate parameters for the fast and slow release components, and *f* is the fraction of drug subject to fast release. Finally, we considered a 3-parameter non-linear model used by the Population Council to describe release rates for Jadelle® [3]:

$L_{i}=A+\beta_{1}t_{i}^{0.5}+\beta_{2}t_{i}$. (4)

Of note, the release rate for model (4) converges to a constant (β_2_) as *t_i_* gets large. Although terminal zero-order release is not plausible, the model provided a reasonable fit to the first 3 years of implant use in a previous report [4].

Parameter estimates along with model fits to the explant data are provided for each of the four prespecified models in Supplemental Table 1. Of note, the bi-exponential model would not converge for the Jadelle® data, possibly due to the scarcity of explants in the first few days and weeks post-insertion.

The *f2* similarity factor is given by

 (5)

where *X****_·_****_Tj_* and *X****_·_****_Rj_* are the average percent release values at time *j* for the test and reference products, respectively, and *p* is the number of assessed time points. Values between 50 (~10% average difference in release) and 100 (perfect agreement) indicate similar dissolution profiles. Computing *f*2 in the current setting required grouping explants into 6-month use intervals, beginning at 3 months and extending through month 51. Guidance indicates that at least 12 observations should be available per time point [5], but this could not be achieved using our monthly sampling strategy. We used the nominal LNG load of 150 mg when computing average percent release in the expression for *f*2.

We assessed the impacts of BMI, age, and race by adding covariates to the regression parameters for the burst fraction and exponential decay. Each variable (BMI, age, and race) was assessed individually alongside the device type covariates for baseline load, burst fraction, and exponential decay. BMI and age were both centered on the mean value over all participants, and race was assessed using the variable for study since all participants in the China study were Asian and almost all participants in the DR study were biracial. P-values were based on likelihood ratio tests with 2 degrees of freedom based on an overall effect for each variable. Neither BMI (p=0.431), age (p=0.364), nor race (p=0.079) were statistically significant. For race, this analysis is limited by the fact that explants from the China study were available only after about 36 months of use and therefore the estimation of the burst fraction required extrapolation 3 years outside of the range of the data.

**References**

1. Creinin MD, Jansen R, Starr RM, et al. Levonorgestrel release rates over 5 years with the Liletta® 52-mg intrauterine system. Contraception 2016; 94:353-56
2. Fu Y and Kao WJ. Drug release kinetics and transport mechanisms of non-degradable and degradable polymeric delivery systems. Expert Opin Drug Deliv 2010; 7:429-444.
3. US FDA new drug application 20-544: Jadelle summary basis of approval. Freedom of Information Act 2008; 2002. p 19.
4. Callahan RL, Taylor D, Jenkins DW, et al. In vivo release of levonorgestrel from Sino-implant (II) – an innovative comparison of explant data. Contraception 2015; 92:350-55.
5. European Medicines Agency Committee for Medicinal Products for Human Use. Guideline on the investigation of bioequivalence; 2010. Accessed at https://www.ema.europa.eu/en/documents/scientific-guideline/guideline-investigation-bioequivalence-rev1_en.pdf.

**Supplemental Table 1.** Model Fits to Explant data

|  | **Parameter Estimates^†^ (SE)** | | | | |  |
| --- | --- | --- | --- | --- | --- | --- |
| **Device**/model | ***A*** | ***f*** | ***k_f_*** | ***k*** | ***σ^2^*** | **BIC^‡^** |
| **Sino-implant (II)** |  |  |  |  |  |  |
| Mono-exponential (M1) | 137.2 (1.3) | NA | NA | 2.49E-4 (1.2E-5) | 1.73E-3 (3.4E-4) | -174.7 |
| Mono-exp. with burst (M2) | 142.8 (2.5) | 0.057 (0.02) | NA | 2.32E-4 (9.8E-6) | 1.54E-3 (2.8E-4) | -212.7 |
| Bi-exponential (M3) | 138.9 (1.7) | 0.513 (0.12) | 6.13E-4 (2.2E-4) | 8.59E-9 (7.7E-6) | 1.65E-3 (3.2E-4) | -169.5 |
|  | ***A*** | ***-*** | ***Β_1_*** | ***Β_2_*** | ***σ^2^*** |  |
| Population Council (M4) | 141.1 (2.1) | - | -0.672 (0.19) | -0.013  (3.8E-3) | 1.65E-3 (3.0E-4) | -208.3 |
| **Jadelle®** |  |  |  |  |  |  |
| Mono-exponential (M1) | 141.8 (1.5) | — | — | 3.09E-4 (1.3E-5) | 2.08E-3 (4.0E-4) | -168.3 |
| Mono-exp. with burst (M2) | 150.5 (2.7) | 0.079 (0.02) | — | 2.87E-4 (1.3E-5) | 1.63E-3 (3.2E-4) | -177.6 |
| Bi-exponential (M3) | *failed to converge* | | | | | - |
|  | ***A*** | ***-*** | ***Β_1_*** | ***Β_2_*** | ***σ^2^*** |  |
| Population Council (M4) | 146.7 (2.4) | — | -0.840 (0.23) | -0.016  (5.2E-3) | 1.89E-3 (3.7E-4) | -169.4 |

^†^*A* ≡ Baseline LNG; *f* ≡ burst fraction (M2) or fast release fraction (M3); *k_f_* ≡ fast release rate (M3); *k* ≡ exponential release rate (M1 and M2) or slow release rate (M3); *σ^2^* ≡ residual (log-scale) variance; *(B_1_,B_2_)* ≡ parameters unique to the Population Council model.

**^‡^** For each device type, model with smallest Bayesian Information Criterion (BIC) is preferred.

1. Corresponding author. Tel.: +1-919-544-7040, ext. 11732; e-mail: rfuchs@fhi360.org [↑](#footnote-ref-2)
